# Determinants of mortality in a large group of hemodialysis patients hospitalized for COVID-19

**DOI:** 10.1186/s12882-021-02233-0

**Published:** 2021-01-14

**Authors:** Kenan Turgutalp, Savas Ozturk, Mustafa Arici, Necmi Eren, Numan Gorgulu, Mahmut Islam, Sami Uzun, Tamer Sakaci, Zeki Aydin, Erkan Sengul, Bulent Demirelli, Yavuz Ayar, Mehmet Riza Altiparmak, Savas Sipahi, Ilay Berke Mentes, Tuba Elif Ozler, Ebru Gok Oguz, Bulent Huddam, Ender Hur, Rumeyza Kazancioglu, Ozkan Gungor, Bulent Tokgoz, Halil Zeki Tonbul, Alaattin Yildiz, Siren Sezer, Ali Riza Odabas, Kenan Ates

**Affiliations:** 1 Department of Nephrology, Mersin University Faculty of Medicine, Mersin, Turkey; 2 Department of Nephrology, Haseki Training and Research Hospital, Istanbul, Turkey; 3 Department of Nephrology, Hacettepe University Faculty of Medicine, Ankara, Turkey; 4 Department of Nephrology, Kocaeli University Faculty of Medicine, Kocaeli, Turkey; 5 Department of Internal Medicine, Division of Nephrology, University of Health Sciences, Bagcilar Training and Research Hospital, Istanbul, Turkey; 6 Department of Nephrology, Zonguldak Ataturk State Hospital, Zonguldak, Turkey; 7 Health Sciences University, Sisli Hamidiye Etfal Education and Research Hospital, Nephrology Department, Istanbul, Turkey; 8 Department of Nephrology, Darica Farabi Training and Research Hospital, Kocaeli, Turkey; 9 Health Science University, Kocaeli Derince Education and Research Hospital, Division of Nephrology, Kocaeli, Turkey; 10 Department of Nephrology, University of Health Sciences, Haydarpasa Numune Education and Research Hospital, Istanbul, Turkey; 11 Department of Nephrology, Bursa City Hospital, Bursa, Turkey; 12 Department of Internal Medicine, Division of Nephrology, Istanbul University- Cerrahpasa Cerrahpasa Faculty of Medicine, Istanbul, Turkey; 13 Department of Internal Medicine, Division of Nephrology, Sakarya University Faculty of Medicine, Education and Research Hospital, Sakarya, Turkey; 14 Department of Internal Medicine, Division of Nephrology, Marmara University School of Medicine. Marmara University Pendik Education and Research Hospital, Istanbul, Turkey; 15 Department of Internal Medicine, Division of Nephrology, Kanuni Sultan Suleyman Research and Training Hospital, Istanbul, Turkey; 16 Department of Nephrology, University of Health Sciences, Diskapi Yildirim Beyazit Education and Research Hospital, Ankara, Turkey; 17 Departmant of Internal Medicine, Division of Nefrology, Mugla Sitki Kocman University Faculty of Medicine, Education and Research Hospital, Mugla, Turkey; 18 Department of Nephrology, Manisa Merkezefendi State Hospital, Manisa, Turkey; 19 Department of Internal Medicine, Division of Nephrology, Bezmialem Vakif University, Istanbul, Turkey; 20 Department of Nephrology, Kahramanmaras Sutcu Imam University, Faculty of Medicine, Kahramanmaras, Turkey; 21 Erciyes University School of Medicine Department of Nephrology, Kayseri, Turkey; 22 Department of Internal Medicine, Division of Nephrology, Necmettin Erbakan University, Konya, Turkey; 23 Department of Internal Medicine, Division of Nephrology, Istanbul University, Istanbul School of Medicine, Istanbul, Turkey; 24 Department of Internal Medicine and Nephrology, Atılım University Faculty of Medicine, Ankara, Turkey; 25 Department of Nephrology, University of Health Sciences, Sultan Abdulhamid Han Training and Research Hospital, Istanbul, Turkey; 26 Ankara University Faculty of Medicine Department of Nephrology, Ankara, Turkey

**Keywords:** Hemodialysis, COVID-19, Radiological manifestations, Mortality, Clinical findings

## Abstract

**Background:**

Maintenance hemodialysis (MHD) patients are at increased risk for coronavirus disease 2019 (COVID-19). The aim of this study was to describe clinical, laboratory, and radiologic characteristics and determinants of mortality in a large group of MHD patients hospitalized for COVID-19.

**Methods:**

This multicenter, retrospective, observational study collected data from 47 nephrology clinics in Turkey. Baseline clinical, laboratory and radiological characteristics, and COVID-19 treatments during hospitalization, need for intensive care and mechanical ventilation were recorded. The main study outcome was in-hospital mortality and the determinants were analyzed by Cox regression survival analysis.

**Results:**

Of 567 MHD patients, 93 (16.3%) patients died, 134 (23.6%) patients admitted to intensive care unit (ICU) and 91 of the ones in ICU (67.9%) needed mechanical ventilation. Patients who died were older (median age, 66 [57–74] vs. 63 [52–71] years, *p* = 0.019), had more congestive heart failure (34.9% versus 20.7%, *p* = 0.004) and chronic obstructive pulmonary disease (23.6% versus 12.7%, *p* = 0.008) compared to the discharged patients. Most patients (89.6%) had radiological manifestations compatible with COVID-19 pulmonary involvement. Median platelet (166 × 10^3^ per mm^3^ versus 192 × 10^3^ per mm^3^, *p* = 0.011) and lymphocyte (800 per mm^3^ versus 1000 per mm^3^, *p* < 0.001) counts and albumin levels (median, 3.2 g/dl versus 3.5 g/dl, *p* = 0.001) on admission were lower in patients who died. Age (HR: 1.022 [95% CI, 1.003–1.041], *p* = 0.025), severe-critical disease clinical presentation at the time of diagnosis (HR: 6.223 [95% CI, 2.168–17.863], *p* < 0.001), presence of congestive heart failure (HR: 2.247 [95% CI, 1.228–4.111], *p* = 0.009), ferritin levels on admission (HR; 1.057 [95% CI, 1.006–1.111], *p* = 0.028), elevation of aspartate aminotransferase (AST) (HR; 3.909 [95% CI, 2.143–7.132], p < 0.001) and low platelet count (< 150 × 10^3^ per mm^3^) during hospitalization (HR; 1.864 [95% CI, 1.025–3.390], *p* = 0.041) were risk factors for mortality.

**Conclusion:**

Hospitalized MHD patients with COVID-19 had a high mortality rate. Older age, presence of heart failure, clinical severity of the disease at presentation, ferritin level on admission, decrease in platelet count and increase in AST level during hospitalization may be used to predict the mortality risk of these patients.

## Background

Maintenance hemodialysis (MHD) patients are at increased risk for several infections associated with high mortality. The coronavirus disease 2019 (COVID-19) affected all countries and territories around the world, especially the elderly population and patients with multiple morbidities, including chronic kidney disease. MHD patients constitute a vulnerable population due to several reasons. Most MHD patients are elderly and have a burden of comorbidities, mainly diabetes mellitus, hypertension, or cardiovascular disease [1]. MHD patients have a high risk of exposure to infections because most of them have been treated as in-center patients. It was not feasible for MHD patients to carry out social isolation rules to prevent exposure as they need to travel three times a week to the hemodialysis centers [2]. They have contact with other patients and the staff of the dialysis centers. Moreover, immune dysregulation and immune senescence of uremia increase the vulnerability of MHD patients for COVID-19 [3]. MHD patients are at high risk for COVID-19 related mortality [4]. There is a need for early diagnosis and a timely start to treatment in order to reduce morbidity and mortality in this group [4].

There is limited information about the impact of COVID-19 on the clinical outcomes of MHD patients. Most of the published studies were single-center and consisting of heterogenous inpatient and outpatient populations with small numbers of patients (usually 50 to 100) [5–8]. A recent US study has evaluated the largest number of hospitalized dialysis patients with a majority of hemodialysis patients [9]. There is still not enough information regarding the outcomes of hospitalized COVID-19 MHD patients from different regions/countries. We herein present our experience in Turkey in a larger group (> 500) of hospitalized hemodialysis patients. In this multicenter study, we described the clinical, laboratory, and radiologic characteristics and the factors that determine in-hospital mortality in hospitalized MHD patients for COVID-19.

## Methods

### Study design and subjects

This was a multicenter, retrospective, observational study, and data were collected from 47 nephrology clinics from different regions in Turkey between 17th April to 1st June 2020. All authors transferred their data to the database. Permission was received from all authors to use their data. The main database included hospitalized COVID-19 patients with and without kidney diseases (Stage 3 to 5 chronic kidney disease, dialysis and kidney transplantation patients) [10]. The data of 551 hemodialysis patients in this database were shared as two separate files with ERACODA, which is a European Renal Association-European Dialysis and Transplant Association (ERA-EDTA) database that collects the data of kidney patients with COVID-19. This study, which has an ethics committee number of 41–2020, was approved by the Health Sciences University Istanbul Haseki Training and Research Hospital Ethics Committee. Informed consent from patients was waived due to the need for rapid data collection during the pandemic period by the institutional review board. The research was supported by the Turkish Society of Nephrology without any conditions.

The study had only included hospitalized MHD patients with a COVID-19 diagnosis. MHD patients who were younger than 18 years of age, who lack hospital discharge or survival information, who were still hospitalized during data collection, and patients who hospitalized due to other reasons were excluded from the study.

### COVID-19 diagnosis

Reverse transcription polymerase chain reaction (RT-PCR) testing from nasopharyngeal swab was performed for COVID-19 detection. A positive result was accepted as the evidence for severe acute respiratory syndrome coronavirus 2 (SARS-CoV-2). Patients whose first test was negative but positive in the second test were also considered as confirmed cases. Because the test may have false negative results or may not be available immediately for some patients [11], patients clinically suspected having COVID-19 were mostly screened with a chest computed tomography (CT). If there were radiological findings compatible with COVID-19 pneumonia, the patient was diagnosed as ‘likely case of COVID-19’ as stated by the Ministry of Health “COVID-19 Diagnosis and Treatment Guideline” [11], which is similar to the diagnostic criteria of the European Centre for Disease Prevention and Control [12].

Disease severity was defined according to the clinical presentation of COVID-19 patients: Mild disease defines patients with mild clinical symptoms without a findings of viral pneumonia on chest CT. Moderate disease defines patients with symptoms like fever and cough, dyspnea and signs of viral pneumonia on chest CT findings; severe disease refers to patients with any of the following criteria: respiratory rate ≥ 30 breaths/min, resting oxygen saturation ≤ 93%; arterial PO2/FiO2 ≤ 300 mmHg. Patients with pulmonary lesion growth rate > 50% within 24–48 h on radiologic imaging were also defined as severe diseases. Patients with respiratory failure needing mechanical ventilation, shock, multiorgan failure or need intensive care unit (ICU) follow-up were defined as having critical disease [11].

### Data collection and outcome

In all centers, data were collected by reviewing electronic health records of the hospitals. Baseline data have included symptoms (such as fever, fatigue, dry cough, dyspnea, diarrhea, anorexia, myalgia, headache and sore throat), comorbidities and medications, initial laboratory assessments [serum creatinine, albumin, ferritin, C-reactive protein (CRP), haemoglobin, lymphocyte and platelet count, aspartate aminotransferase (AST) and lactate dehydrogenase levels (LDH)]. The location of the hemodialysis sessions were recorded.

Dataset also included the disease severity of COVID-19, laboratory tests performed during hospitalization and medications that were given for COVID-19 treatment (hydroxychloroquine, oseltamivir, macrolides, lopinavir-ritonavir, favipiravir, glucocorticoids, tocilizumab, convalescent plasma, anakinra). Patients’ need for ICU care and mechanical ventilation were also noted. The main study outcome was defined as in-hospital mortality.

### Statistical analysis

The IBM SPSS Statistics for Windows, Version 25.0 (IBM Corp., Armonk, NY, USA) was used for statistical analysis. Numerical variables were expressed as median and 25th percentile-75th percentile; categorical variables were expressed as numbers and percentages. Student’s t-test was used for normal distributed data and, Mann-Whitney U test was used for abnormally distributed data in the comparison of numerical parameters of dead and discharged patients. Chi-square test was used to compare the categorical data. To find out the independent parameters related to the mortality, we used a multivariate Cox regression model (Backward LR method) including the parameters found related to survival in the univariate analyses. *p*-value < 0.05 was considered significant.

## Results

**Fig. 1:**
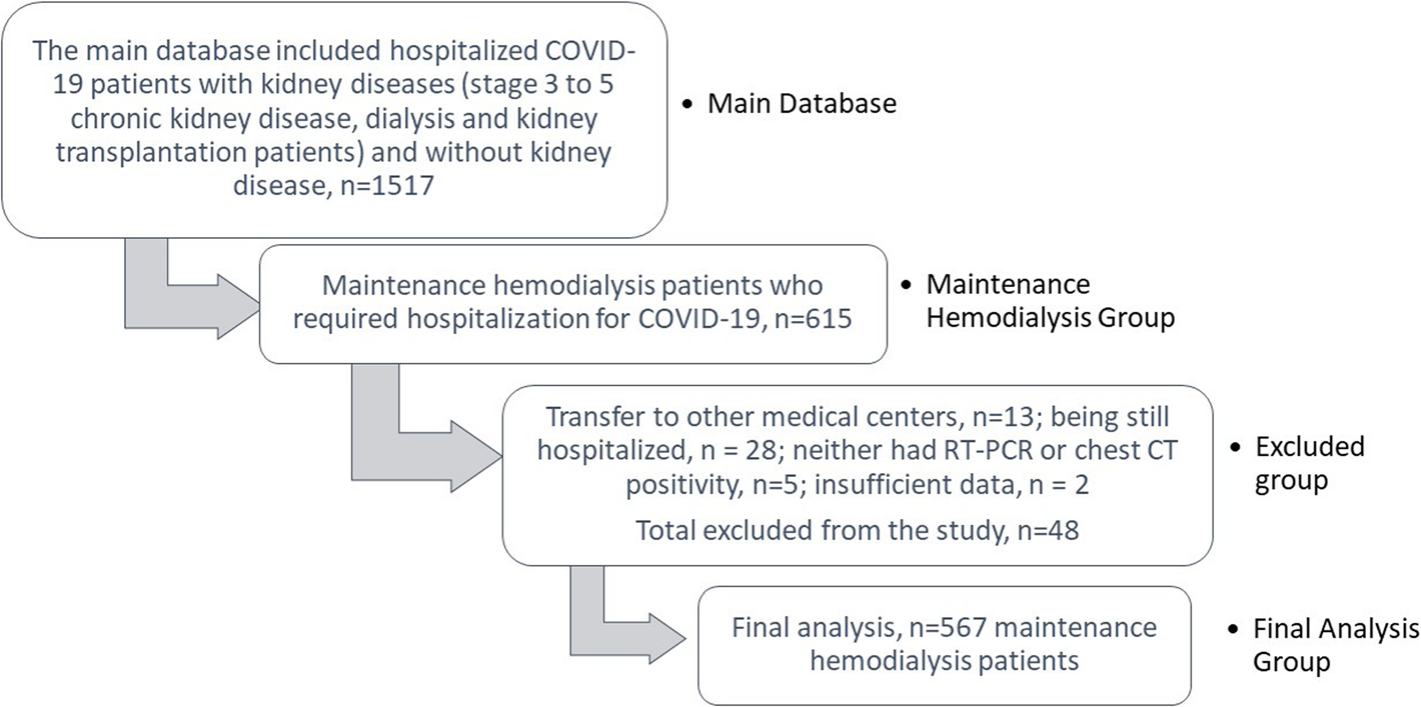
Flow chart illustrating the study population selection

The study was conducted from 17th April to 1st June 2020 and included 615 MHD patients who required hospitalization for COVID-19. 48 patients were excluded due to various reasons: transfer to other medical centers (*n* = 13), being still hospitalized (*n* = 28), neither had RT-PCR or chest CT positivity for COVID-19 diagnosis (*n* = 5) and insufficient data (n = 2). The final analysis was done in 567 MHD patients [296 male (52.2%)]. The study population selection was shown in Fig. 1.

### Clinical characteristics and laboratory findings

Baseline demographic and laboratory characteristics of the patients were shown in Table 1. The median age was 63 (interquartile range [IQR], 53–71) years. The prevalence of comorbid conditions such as hypertension, cardiovascular diseases, diabetes mellitus, congestive heart failure, chronic obstructive pulmonary disease (COPD) were found as 79.1, 49.3, 46.5, 23, 14.6% respectively. The median duration of hemodialysis treatment was 3 years (IQR 1–6).

**Table 1:** Baseline demographic and laboratory characteristics of maintenance hemodialysis patients

		Dead (*n* = 93)	Discharged (*n* = 474)	All (*n* = 567)	*p* value*
Age (years), median (IQR)		66 (57-74)	63 (52-71)	63 (53-71)	*0.019*
Hemodialysis duration (year), median		4.0 (1.8-6.0)	3.0 (1.0-6.0)	3.0 (1.0-6.0)	0.326
Gender n (%)	Female	39 (41.9)	232 (48.9)	271 (47.8)	0.196
	Male	54 (58.1)	242 (51.1)	296 (52.2)	
Symptoms on admission n (%)					
	Fever	50 (53.8)	257 (53.9)	307 (53.9)	0.984
	Dyspnea	78 (83.9)	229 (48.0)	307 (53.9)	*<0.001*
	Dry cough	53 (57.0)	206 (43.2)	259 (45.4)	*0.014*
	Fatigue	51 (54.8)	184 (38.6)	235 (41.2)	*0.004*
	Myalgia	25 (26.9)	77 (16.1)	102 (17.9)	*0.013*
	Headache	9 (9.7)	37 (7.8)	46 (8.1)	0.534
	Sore throat	8 (8.6)	37 (7.8)	45 (7.9)	0.076
	Anorexia	12(12.9)	25(5.2)	37 (6.5)	*0.006*
	Diarrhea	7 (7.5)	15 (3.1)	22 (3.9)	*0.045*
	No symptom	1(1.1)	42 (8.8)	43 (7.5)	*0.01*
COVID-19 Diagnosis					
Positive nasopharyngeal swap PCR, n (%)		57 (61.3)	214 (45.1)	271 (47.7)	*0.004*
Radiological manifestations n (%)		88 (94.6)	423 (88.7)	511 (89.6)	0.085
CT findings n (%)	Bilateral multiple lesions	75 (80.6)	326 (68.3)	401 (70.4)	*0.045*
	Unilateral multiple lesions	8 (8.6)	44 (9.2)	52 (9.1)	
	Soliter lesion	3 (3.2)	42 (8.8)	44 (7.7)	
	Completely normal	0 (0)	25 (5.2)	25 (4.4)	
Time between first symptom-diagnosis (days), Median (IQR)		3 (2-5)	3 (1-4)	3 (2-4)	0.091
COVID-19 disease severity, n (%)	Mild-Moderate Disease	11 (11.8)	286 (60.3)	297 (52.4)	*0.001*
	Severe-Critical Disease	82 (88.2)	188 (39.7)	270 (47.6)	
Laboratory findings, on admission					
	Creatinine (mg/dl)	6.95 (5-8.33)	6.79 (5.3-8.77)	6.79 (5.2-8.7)	0.353
	Albumin (g/dl)	3.2 (2.87-3.6)	3.5 (3.1-3.85)	3.40 (3.08-3.8)	*0.001*
	Ferritin (ng/L)	987 (520.5-2000)	870 (500-1429)	883 (500-1500)	*0.044*
	Haemoglobin (g/dl)	10.4 (9.4-11.6)	10.4 (9-11.8)	10.4 (9.1-11.8)	0.80
	Lymphocyte count (mm^3^)	800 (450-980)	1000 (750-1380)	970 (660-1352)	*<0.001*
	Platelet count (x1000/mm^3^)	166 (130-232)	192 (151-246)	186 (147-245)	*0.011*
	CRP≥10 (x of upper normal value), n (%)	72 (77.4)	215 (45.1)	287 (50.4)	*<0.001*
Laboratory findings, during hospitalization, n (%)					
	LDH (>2×upper limit of normal)	52 (60.5)	85 (19.0)	137 (25.7)	*<0.001*
	AST (>2×upper limit of normal)	44 (49.4)	31 (6.6)	75 (13.5)	
	Lymphopenia (<1500/mm^3^)	80 (88.9)	264 (56.1)	344 (61.3)	
	Thrombocytopenia (<150x10^3^/mm^3^)	39(42.4)	102(21.7)	141 (25.0)	
	Leukopenia (<4000/mm^3^)	25 (27.5)	90 (19.1)	115 (20.4)	0.06
	Anemia (Hgb<10 g/dL)	61 (66.3)	230 (49.5)	291 (52.2)	*0.003*
Coexisting disorder, n (%)					
	Diabetes mellitus	43 (47.3)	218 (46.4)	261 (46.5)	0.879
	Hypertension	70 (79.5)	374 (79.1)	444 (79.1)	0.920
	Ischemic heart disease	42(49.4)	180 (42.0)	222 (43.2)	0.205
	Cardiovascular disease	48 (55.2)	208 (48.1)	256 (49.3)	0.232
	Congestive heart failure	30 (34.9)	90 (20.7)	120 (23.0)	*0.004*
	COPD	21(23.6)	56 (12.7)	77 (14.6)	*0.008*
	Malignancy	6 (6.5)	24 (5.3)	30 (5.5)	0.650
Outcomes					
Length of stay at hospital (days), median (IQR)		8 (5-14)	10 (7-14)	9 (6-12)	0.856
ICU admission, n (%)	Yes	89 (96.7)	45 (9.5)	134 (23.6)	*<0.001*
	No	3 (3.3)	430 (90.5)	433 (76.4)	
Mechanical ventilation in ICU, n (%)	Yes	86 (97.7)	5 (11.6)	91 (69.5)	*<0.001*
	No	2 (2.3)	38 (88.4)	40 (30.5)	

*Abbreviations*: *IQR* interquartile range, *COVID-19* coronavirus disease 2019, *CRP* C-reactive protein, *LDH* lactate dehydrogenase, *AST* aspartate aminotransferase, *Hgb* Haemoglobin, *HD* hemodialysis, *ICU* intensive care unit, *CT* computed tomography, *COPD* chronic obstructive pulmonary disease

*p value pertains to comparing subsets of dead versus discharged groups

The median time between the onset of the first symptom of the COVID-19 and diagnosis was 3 days (IQR 2–4). The most common symptoms on admission were fever (53.9%), dyspnea (53.9%), dry cough (45.4%), fatigue (41.2%), myalgia (17.9%), headache (8.1%), sore throat (7.9%), anorexia (6.5%), diarrhea (3.9%), and 7.5% of the patients had no symptoms. 56.8% of patients were never smokers, 30.9% of patients were former smokers and 12.3% of patients were current smokers.

Most of the patients had anemia and lymphopenia on admission. While median levels of albumin were also at lower ranges [3.40 g/dL, (3.08–3.8)], ferritin [883 ng/L (500–1500)], levels were high for the majority of the patients (Table 1). The median CRP level was elevated in almost all patients (93.5%).

More than half of the patients (*n* = 297, 52.4%) were admitted with a mild-moderate clinical presentation, but 270 (47.6%) patients were in severe-critical state. Baseline characteristics and laboratory findings of patients according to the disease severity were shown in Table 2. The majority of the patients with mild-moderate disease had no symptoms compared to patients with severe-critical disease (*p* < 0.001). Dyspnea was the most common symptom in patients with severe-critical disease (p < 0.001). In severe-critical patients, LDH (> 2 × upper limit of normal), AST (> 2 × upper limit of normal), lymphopenia (< 1500/mm^3^) and thrombocytopenia (< 150 × 10^3^/mm^3^) rates during hospitalization were significantly higher compared to patients with mild-moderate disease (for all, p < 0.001).

**Table 2:** Baseline characteristics and laboratory findings of MHD patients with COVID-19 according to the disease severity

	Mild-moderate disease (n = 297)	Severe-critical disease (*n* = 270)	p value
Age (year)	63 (52.00–71.00)	64 (54.00–73.00)	0.26
Hemodialysis duration (years)	3 (1.40–6.00)	3 (1.00–6.00)	0.86
**LDH (> 2 × upper limit of normal), DH**	**47 (17.2)**	**89 (34.5)**	***< 0.001***
**AST (> 2 × upper limit of normal), DH**	**17 (5.9)**	**58 (21.9)**	***< 0.001***
**Lymphopenia (< 1500/mm**^**3**^**), DH**	**154 (52.7)**	**189 (71.1)**	***< 0.001***
**Thrombocytopenia (< 150 × 10**^**3**^**/mm**^**3**^**), DH**	**56 (19.2)**	**85 (31.6)**	***< 0.001***
Fever, n (%)	153 (51.5)	153 (56.7)	0.21
**Dyspnea, n (%)**	**90 (30.3)**	**217 (80.4)**	***< 0.001***
Dry cough, n (%)	129 (43.4)	130 (48.1)	0.26
Fatigue, n (%)	112 (37.7)	123 (45.6)	0.58
Myalgia, n (%)	50 (16.8)	52 (19.3)	0.45
Headache, n (%)	24 (8.1)	22 (8.1)	0.97
Sore throat, n (%)	25 (8.4)	20 (7.4)	0.65
**Anorexia, n (%)**	**13 (4.4)**	**24 (8.9)**	***0.030***
Diarrhea, n (%)	13 (4.4)	9 (3.3)	0.52
**No symptom, n (%)**	**40 (13.5)**	**2 (0.7)**	***< 0.001***
ICU admission, n (%)	19 (6.4)	115 (42.5)	< 0.001
Mechanical ventilation need, n (%)*	10 (52.6)	81 (72.3)	0.08
In-hospital mortality, n (%)	11 (3.7)	82 (30.4)	< 0.001

**Abbreviations: COVID-19,** coronavirus disease 2019; **LDH,** lactate dehydrogenase; **DH,** during hospitalization; **AST,** aspartate aminotransferase; **ICU,** intensive care unit

*The percentage among ICU admitted patients

The most commonly used medications at the admission were erythropoiesis-stimulating agents (76.5%), vitamin D receptor analogues (59.3%), beta-blockers (46.9%), calcium channel blockers (44.4%), insulin (38%) and renin-angiotensin-aldosterone system (RAAS) blockers (27.6%).

The drugs that patients received for COVID-19 treatment in the hospital were chloroquine/hydroxychloroquine (96.6%), macrolides (78.7%), oseltamivir (60.7%), favipiravir (32.4%), glucocorticoids (4%), lopinavir-ritonavir (1.8%), tocilizumab (1.5%), anakinra (0.9%), convalescent plasma (0.4%).

Dialysis was carried out in ICU, in isolated hospital rooms or in special reserved areas of hospitals’ hemodialysis centers as appropriate.

### Radiologic findings

Almost all patients had radiological manifestations (89.6%) compatible with COVID-19 pulmonary involvement (Table 1). Of all chest CT scans that were performed, the most common abnormalities were ground-glass opacities (82.9%), pleural effusion (24.8%), lymphadenopathy (8.1%), reticular pattern (13%) and vasculitic thickening (4.8%). These lesions were often detected as bilateral multiple lesions (70.4%) compatible with COVID-19 pneumonia. Chest CT findings of COVID-19 MHD patients were shown in Table 1.

### Clinical outcomes

The clinical outcomes of the patients were shown in Table 1. 93 (16.2%) of the total patients who participate in the study died. A total of 134 (23.6%) patients needed ICU care and 91 of them (67.9%) required mechanical ventilation. Mechanical ventilation requirement in the whole group was 16.0%. Patients who died had more ICU and mechanical ventilation need, compared to the patients who were discharged (96.7% versus 9.5 and 97.7% versus 11.6% respectively, *p* < 0.001). There was no gender difference between patients who died or were discharged from the hospital (*p* > 0.05). The median age of the patients who died was higher compared to discharged patients (median age, 66 versus 63 years, *p* = 0.019). Similarly, congestive heart failure (34.9% versus 20.7%, *p* = 0.004) and COPD (23.6% versus 12.7%, *p* = 0.008) frequencies were significantly higher in patients who died compared to the discharged patients.

As shown in Table 1, symptoms like fatigue, dry cough, anorexia, dyspnea, myalgia, and diarrhea were more frequent in patients who died (*p* < 0.05). Median platelet (166 × 10^3^ /mm3 versus 192 × 10^3^ /mm^3^, *p* = 0.011) and lymphocyte (800 /mm^3^ versus 1000 /mm^3^, *p* < 0.001) counts and albumin levels (median, 3.2 g/dL versus 3.5 g/dL, *p* = 0.001) on admission were found lower in patients who died. Furthermore, rates of lymphopenia (< 1500/mm^3^, 88.9% versus 56.1%, *p* < 0.001), thrombocytopenia (< 150 × 10^3^/mm^3^, 42.4% versus 21.7%, p < 0.001), leukopenia (< 4000/mm^3^, 27.5% versus 19.1%, p < 0.001), anemia (haemoglobin< 10 g/dL, 66.3% versus 49.5%, *p* < 0.001), high AST (> 2 × upper limit of normal, 49.4% versus 6.6%, p < 0.001) and high LDH levels (> 2 × upper limit of normal, 60.5% versus 19%, *p* < 0.001) during hospitalization were clearly higher in patients who died compared to the discharged patients. Ferritin and CRP levels were also higher in patients who died (*p* < 0.05 and p < 0.001). There was no difference in smoking habits between patients who died and were discharged (*p* = 0.184).

The rate of severe-critical clinical presentation was higher in patients who died compared to the ones who were discharged (88.2% versus 39.7%, *p* < 0.001). Rate of mortality (30.4% versus 3.7%) and ICU need (42.7% versus 6.4%) were higher in severe-critical patients compared to patients with mild-moderate disease (for all, p < 0.001). The discharge rate of patients with mild-moderate disease was higher than that of patients who have severe-critical disease (96.3% versus 69.6%, *p* < 0.001).

Favipiravir and glucocorticoid usage rates were higher in patients who died in the hospital compared with patients who were discharged (66.7% versus 25.3 and 14.1% versus 2.1% for all p < 0.001). There was not any significant difference between died and discharged group for RAAS blockers, beta-blockers, insulin, statin, oral anti-diabetic, erythropoiesis-stimulating agents, vitamin D receptor analogues, phosphate binders and iron medication use at admission.

Radiological manifestations (89.6%) were similar between patients who died and discharged (94.6% versus 88.7%, *p* > 0.05). RT-PCR positive test rate (47.7%) was significantly higher in patients who died (61.3%) than discharged (45.1%) (*p* < 0.001). Bilateral multiple lesions on chest CT were seen more in patients who had died compared to those who were discharged (80.6% versus 68.3%, *p* < 0.05).

### Factors associated with risk of mortality

Cox regression survival analysis showed that age (HR; 1.022 [95% CI, 1.003–1.041], *p* = 0.025), severe-critical disease at the time of diagnosis (HR; 6.223 [95% CI, 2.168–17.863], p < 0.001), presence of congestive heart failure (HR; 2.247 [95% CI, 1.228–4.111], *p* = 0.009), ferritin levels on admission (HR; 1.057 [95% CI, 1.006–1.111], *p* = 0.028), AST (> 2x upper limit of normal) during hospitalization (HR; 3.909 [95% CI, 2.143–7.132], p < 0.001), thrombocytopenia (< 150 × 10^9^/L) during hospitalization (HR; 1.864 [95% CI, 1.025–3.390], *p* = 0.041) were risk factors for mortality (Table 3).

**Table 3:** Cox analysis of mortality

	HR	95% CI for HR		p
		Lower	Upper	
Age (Year)	1.022	1.003	1.041	0.025
COVID-19 disease severity*	6.223	2.168	17.863	0.001
Congestive heart failure	2.247	1.228	4.111	0.009
AST (> 2 × upper limit of normal) during hospitalization	3.909	2.143	7.132	< 0.001
Thrombocytopenia (< 150 × 10^9^/L) during hospitalization	1.864	1.025	3.390	0.041
Anaemia (Hgb < 10 g/dl) during hospitalization	0.541	0.284	1.029	0.061
CRP (mg/L)**	1.767	0.890	3.507	0.104
Ferritin (ng/L)	1.057	1.006	1.111	0.028

* Severe-Critical Disease/ Mild-Moderate Disease ** ≥10 times increase/ < 10 times increase

**Abbreviations; COVID-19,** coronavirus disease 2019; **AST,** aspartate aminotransferase; **Hgb,** Haemoglobin; **CRP,** C-reactive protein

**The following variables were included in the model, which were found to be associated with mortality in univariate analyses:** Age, gender, COVID-19 related clinic presentation at the time of diagnosis, congestive heart failure, chronic obstructive pulmonary disease, time between first symptom-diagnosis, diagnosis with nasopharyngeal swab PCR test, diagnosis with radiological test, lymphocyte, platelet count, CRP level, serum albumin and ferritin at admission, AST (> 2 × upper limit of normal), LDH (> 2 × upper limit of normal), lymphopenia (< 1.5 × 10^9^ /L), anaemia (Hgb < 10 g/dl) and thrombocytopenia (< 150 × 10^9^/L) during hospitalization, computed tomography (CT) findings

## Discussion

In this multi-center study of a large group of hospitalized MHD patients with COVID-19, we have found a mortality rate of 16.2% and mechanical ventilation need in 16.0% of the patients. Age, presence of congestive heart failure, severe-critical disease, high ferritin levels on admission, high AST levels (> 2 x upper limit of normal) and thrombocytopenia during hospitalization were associated with increased risk for mortality. Patients who had in-hospital mortality had a significantly higher need for ICU care and mechanical ventilation. Although many studies were reported for MHD patients during COVID-19 outbreak, most of the studies had small sample sizes and there was a paucity of data for determinants of hospital mortality. To the best of our knowledge, this is the first study with the highest number of patients, which assessed the short term outcome in hospitalized COVID-19 MHD patients.

It is difficult to implement social isolation measures, prevent and control infectious diseases including COVID-19 in dialysis patients, as they are spending time in crowded waiting areas before and after the hemodialysis sessions [13]. MHD patients have a less efficient immune system that may change their response to COVID-19. Therefore, it is not surprising to see increased mortality rates in MHD patients [14]. As of 12 December 2020, there had been more than 70 million confirmed cases of COVID-19 with 1,588,854 deaths (2.3%) all over the World, reported by the World Health Organization (WHO) [15]. There had been 2.7% mortality rate in hospitalized patients and 4.3% mechanical ventilation need of COVID-19 in the general population in our country according to national data of our Ministry of Health (about 362.800 COVID-19 patients by October 26, 2020) [16]. The mortality rate of hospitalized MHD with COVID-19 patients in our study (16.2%) was 6 times higher than the general population. Previous studies from Italy showed much higher mortality rates in small numbers of hemodialysis patients as 41% (41 patients with a mean age of 73) [17] and 24% (21 patients, mean age not mentioned) [18]. The mortality rate in Spain was also high (30.5%) with a more elderly (mean age 71 years) population [19]. In a study from New York, among 419 hemodialysis and peritoneal dialysis patients (mean age of 74 years) hospitalized with COVID-19, 133 patients died (31.7%) and 89 patients needed mechanical ventilation (21.2%) [9]. In these studies, mortality rate and mechanical ventilation requirements were higher than our study. This large discrepancy between mortality and mechanical ventilation rates may be explained by the major difference of median age of the patients. The mortality rate of COVID-19 MHD patients in all these studies was higher than the general population. This difference may easily be explained by the presence of multiple comorbid conditions such as high cardiovascular comorbidity in patients with MHD as well as being more older than the other cohorts. Our results also showed that patients who died were much older compared to discharged patients that is similar to Valeri et al.’s observations [4].

The median time between the occurrence of first symptoms and diagnosis of COVID-19 was three days in this study. This implies that most patients had been hospitalized in less than a week after their symptoms have started. Moreover, MHD patients diagnosed with COVID-19 during the pandemic were hospitalized at the discretion of the treating physician considering the accompanying risk factors such as advanced age, comorbidities, having dialysis in a remote center, and not having a private transfer opportunity to a dialysis center. Therefore, even a group of asymptomatic patients (7.5%) were hospitalized in this cohort. In most studies, time to diagnosis had not been reported. The length of hospital stay was reported as 11.4 days by Goicoechea et al. [19] and as 12 days by Alberici et al. [18]. In both studies, the length of hospital stay was higher than our study. This difference may be attributed to early diagnosis and prompt hospitalization of our patients. Early diagnosis and hospitalization may also ensure timely management of COVID-19 symptoms and institution of therapeutic approaches. This may also conduce to earlier discharge rates in our study compared to earlier reports. Another reason for the short median length of hospital stay may be the high number of mild-moderate patients in our study according to Goicoechea et al. and Alberici et al. study.

Clinical presentations of COVID-19 in MHD patients were highly variable. In our country, the pandemic plan prepared by Ministry of Health has separated patients into groups as severe-critical or mild-moderate for better management and timely treatment of the severe cases [11]. In this study, most of the symptoms on admission were fever, dyspnea, dry cough, fatigue, whereas diarrhea, anorexia or sore throat was rare. The most common symptom in Goicoechea et al. [19] and Wang et al. studies’ [20] was fever (67 and 60%). During admission, the clinical symptoms of more than half of the patients were mild-moderate and a small proportion had no symptoms. The rate of severe-critical disease rate was 47.6% in this study. Xiong et al. found rate of severe-critical disease in MHD patients as 23% [21], but they did not report the rate of mortality and ICU need. In our study, most of the patients who died had severe-critical presentation. The mortality rate and the ICU need were higher in severe-critical disease as expected. Patients with severe-critical disease had more laboratory evidence indicating presence of a cytokine storm. If COVID-19 infected MHD patients have a severe-critical disease presentation at the time of diagnosis, the mortality risk is 8.2 times higher than the mild-moderate clinical presentation in our cohort.

The severity of viral infection can lead to a decrease in some blood count parameters as markers of mortality. Lymphopenia is one of the most common disorder in COVID-19 and may be an early prognostic indicator [1]. Cytokine storm causing lymphopenia, leukopenia, and high CRP levels were associated with COVID-19 severity [22]. Cytokine storm was closely similar to secondary hemophagocytic lymphohistiocytosis. Secondary hemophagocytic lymphohistiocytosis is usually caused by viral infections and characterized by constant fever and increased ferritin levels [23]. High AST and LDH levels that occur in the cytokine storm indicated that liver dysfunction might have been involved [24]. Furthermore, the cytokine storm is causing hypercoagulability of blood and finally give rise to thrombocytopenia. Thrombocytopenia at admission was an independent risk factor for in-hospital mortality and was associated with almost three-fold increased risk for mortality compared to those without thrombocytopenia [25]. Haemoglobin concentration is one of the most important determinants of the oxygen-binding capacity of the blood. COVID-19 interaction with haemoglobin molecule can cause hemolysis and decrease haemoglobin levels. If the haemoglobin level is low, patients cannot support their increased peripheral tissue demand for oxygen [26]. In our study, haemoglobin levels and lymphocyte counts were found low, ferritin and CRP levels were found high on admission. Higher AST and LDH levels were observed during hospitalization. Patients who died had lower haemoglobin, lymphocyte and platelet counts compared to discharged patients. Baseline CRP levels were found higher in patients who died. This result is consistent with findings in the MHD population reported by Goicoechea et al. [19]. In general population studies, COVID-19 disease mortality was associated with lymphopenia, thrombocytopenia, and elevated CRP levels [27, 28]. In the MHD specific COVID-19 study, the mortality rate has been reported to be associated with high CRP levels [29]. Ng et al. showed in a large group of hemodialysis patients that the mortality risk was associated with increased age, mechanical ventilator, lymphopenia, blood urea nitrogen, and serum ferritin levels [9]. In our study, several laboratory findings, especially, thrombocytopenia and high AST level during hospitalization in COVID-19 MHD patients, were found as risk factors of mortality. In addition, age and ferritin levels were found to be associated with mortality, similar to Ng et al. study [9].

In our study, most patients presented with a radiological abnormality, including multiple bilateral lesions. The majority of our patients were diagnosed with chest CT. RT-PCR test for COVID-19 was not positive in all study patients. However, even if the negative RT-PCR test, typical lesions on chest CT with clinical manifestations were considered as diagnosis of COVID-19. In multivariate analysis, both the RT-PCR test and chest CT were not related to mortality. We think that chest CT may be more valuable for diagnosing COVID-19 in MHD patients. Fang et al. found that the sensitivity of chest CT for COVID-19 diagnosis was greater than RT-PCR (98% versus 71%) [30]. Clinical sensitivity of RT-PCR tests ranges from 66 to 80% in the general population [31]. RT-PCR testing conditions and processes may be far from perfect. That means nearly one in three infected people who are tested will receive false-negative results in the general population. Similar to the general population, RT-PCR test sensitivity may be lower in MHD patients for the diagnosis of COVID-19.

Congestive heart failure is highly prevalent and is a leading cause of mortality in MHD patients [32]. In our study, COPD and congestive heart failure were seen more in patients who died. Classical cardiovascular risk factors in the general population were not determined as risk factors in our study. It was surprising that only congestive heart failure was associated with increased COVID-19 mortality. Alberici et al. reported that many MHD patients had certain comorbid conditions such as cardiovascular disease, hypertension, diabetes, and lung disease, which were related to worse outcomes in patients with COVID-19 [18].

Although our study included a large number of patients from different centers, it has several limitations. Clinical information of patients following their discharge was not obtained, so we could not evaluate the effects of COVID-19 on long-term outcomes. Blood pressure was not included in the dataset. Immunoglobulin M (IgM) and Immunoglobulin G (IgG) antibody seropositivity were not evaluated. The dataset did not include Interleukin 6 (IL-6) or D-Dimer levels, which might be associated with COVID-19 severity and mortality. Height and weight were not measured for most of the patients due to pandemic chaos and severe-critical conditions of many patients. The Sequential Organ Failure Assessment (SOFA) scores, extracorporeal membrane oxygenation (ECMO), continuous renal replacement therapies, vasopressor use, non-invasive ventilation and high flow oxygen need in intensive care were not included in the dataset.

## Conclusion

This study has shown that COVID-19 hospitalized MHD patients had a high mortality rate. Advanced age, presence of heart failure, clinical severity of disease at presentation, ferritin level on admission, platelet count and AST level during hospitalization may be used to predict the mortality risk of these patients.

## Data Availability

The datasets used and/or analysed during the current study available from the corresponding author on reasonable request.

## Abbreviations

AST: asparatat aminotransferase
AV: arteria-venous
COPD: chronic obstructive pulmonary disease
COVID-19: coronavirus disease-2019
CRP: C-reactive protein
CT: computed tomography
DH: during hospitalization
ECMO: extracorporeal membrane oxygenation
ERACODA: European Renal Association-European Dialysis and Transplant Association database
ERA-EDTA: European Renal Association-European Dialysis and Transplant Association
SARS-CoV-2: severe acute respiratory syndrome coronavirus 2
HD: hemodialysis
HR: hazard ratio
95% CI: 95% confidence interval
ICU: intensive care unit
IgG: Immunoglobulin G
IgM: Immunoglobulin M
IL-6: Interleukin 6
IQR: interquartile range
LDH: lactat dehyrogenase
MHD: maintenance hemodialysis
RAAS: renin-angiotensin-aldosterone system
RT-PCR: transcription polymerase chain reaction
SOFA: sequential organ failure assessment
WHO: World Health Organization

## References

[1] Guan WJ, Ni Z, Hu Y, Liang W-H, Ou C-Q, He J-X (2020). Clinical characteristics of coronavirus disease 2019 in China. N Engl J Med.

[2] Naicker S, Yang CW, Hwang SJ, Liu BC, Chen JH, Jha V (2020). The novel coronavirus 2019 epidemic and kidneys. Kidney Int.

[3] Syed-Ahmed M, Narayanan M (2019). Immune dysfunction and risk of infection in chronic kidney disease. Adv Chronic Kidney Dis.

[4] Valeri AM, Robbins-Juarez SY, Stevens JS, Ahn W, Rao MK, Radhakrishnan J (2020). Presentation and outcomes of patients with ESKD and COVID-19. J Am Soc Nephrol.

[5] Zhang J, Cao F, Wu SK, Heng LX, Li W, Li GS (2020). Clinical characteristics of 31 hemodialysis patients with 2019 novel coronavirus: a retrospective study. Ren Fail.

[6] Trivedi M, Shingada A, Shah M, Khanna U, Karnik ND, Ramachandran R. Impact of COVID-19 on maintenance haemodialysis patients: The Indian scenario. Nephrology. (Carlton) 2020; 25(12):929–32. 10.1111/nep.13760 32713060

[7] Wang R, He H, Liao C, Hu H, Hu C, Zhang J (2020). Clinical outcomes of hemodialysis patients infected with severe acute respiratory syndrome coronavirus 2 and impact of proactive chest computed tomography scans. Clin Kidney J.

[8] Roper T, Kumar N, Lewis-Morris T, Moxham V, Kassimatis T, Game D (2020). Delivering Dialysis during the COVID-19 outbreak: strategies and outcomes. Kidney Int Rep..

[9] Ng JH, Hirsch JS, Wanchoo R, Sachdeva M, Sakhiya V, Hong S (2020). Outcomes of patients with end-stage kidney disease hospitalized with COVID-19. Kidney Int.

[10] Ozturk S, Turgutalp K, Arici M, Odabas AR, Altiparmak MR, Aydin Z (2020). Mortality analysis of COVID-19 infection in chronic kidney disease, haemodialysis and renal transplant patients compared with patients without kidney disease: a nationwide analysis from Turkey. Nephrol Dial Transplant.

[11] https://hsgm.saglik.gov.tr/depo/covid19/Ingilizce/Rehber/COVID-19_Rehberi__Ar_pnomoni_ARDS_sepsis_ve_septik_ok_yonetimi_8.06.2020_eng.pdf

[12] https://www.ecdc.europa.eu/en/COVID-19/surveillance/case-definition

[13] Park HC, Lee YK, Lee SH, Yoo KD, Jeon HJ, Ryu DR (2017). Middle East respiratory syndrome clinical practice guideline for hemodialysis facilities. Kidney Res Clin Pract.

[14] Collins AJ, Kliger AS (2018). Urgent: stop preventable infections now. Clin J Am Soc Nephrol.

[15] https://www.who.int/emergencies/diseases/novel-coronavirus-2019?gclid=CjwKCAiA_Kz-BRAJEiwAhJNY71bEwmKrdyUeQB7Gl3LZi1JYE2AmbjgvkkzfiK2DvSd8ya8nqDV0KRoCWHUQAvD_BwE

[16] https://covid19.saglik.gov.tr/Eklenti/39230/0/covid-19-weekly-situation-report%2D%2D-43pdf.pdf?_tag1=D3D202441F1F5165A33D16981E6544EF7FC0A32F

[17] Scarpioni R, Manini A, Valsania T, Amicis SD, Albertazzi V, Melfa L (2020). Covid-19 and its impact on nephropathic patients: the experience at Ospedale “Guglielmo da Saliceto” in Piacenza. G Ital Nefrol.

[18] Alberici F, Delbarba E, Manenti C, Econimo L, Valerio F, Pola A (2020). Management of patients on Dialysis and with kidney transplant during SARS-COV-2 (COVID-19) pandemic in Brescia, Italy. Kidney Int Rep.

[19] Goicoechea M, Sánchez Cámara LA, Macías N, Morales AM, Rojas AG, Bascuñana A (2020). COVID-19: clinical course and outcomes of 36 hemodialysis patients in Spain. Kidney Int.

[20] Wang R, Liao C, He H, Hu C, Wei Z, Hong Z (2020). COVID-19 in hemodialysis patients: a report of 5 cases. Am J Kidney Dis.

[21] Xiong F, Tang H, Liu L, Tu C, Tian JB, Lei CT (2020). Clinical characteristics of and medical interventions for COVID-19 in hemodialysis patients in Wuhan. China J Am Soc Nephrol.

[22] Huang C, Wang Y, Li X, Ren L, Zhao J, Hu Y (2020). Clinical features of patients infected with 2019 novel coronavirus in Wuhan, China. Lancet.

[23] Mehta P, McAuley DF, Brown M, Sanchez E, Tattersall RS, Manson JJ (2020). COVID-19: consider cytokine storm syndromes and immunosuppression. Lancet..

[24] Cai Q, Huang D, Yu H, Zhu Z, Xia Z, Su Y (2020). COVID-19: abnormal liver function tests. J Hepatol.

[25] Liu Y, Sun W, Guo Y, Chen L, Zhang L, Zhao S, Long D, Yu L (2020). Association between platelet parameters and mortality in coronavirus disease 2019: retrospective cohort study. Platelets..

[26] Cavezzi A, Troiani E, Corrao S (2020). COVID-19: hemoglobin, iron, and hypoxia beyond inflammation. A narrative review. Clin Pract.

[27] Zhou F, Yu T, Du R, Fan G, Liu Y, Liu Z (2020). Clinical course and risk factors for mortality of adult inpatients with COVID19 in Wuhan, China: a retrospective cohort study. Lancet..

[28] Zhang G, Hu C, Luo L, Fang F, Chen Y, Li J (2020). Clinical characteristics of 3062 COVID-19 patients: a meta-analysis. J Med Virol.

[29] Trujillo H, Caravaca-Fontán F, Sevillano Á, Gutiérrez E, Caro J, Gutiérrez E (2020). SARS-CoV-2 infection in hospitalized patients with kidney disease. Kidney Int Rep.

[30] Fang Y, Zhang H, Xie J, Lin M, Ying L, Pang P (2020). Sensitivity of chest CT for COVID-19: comparison to RT-PCR. Radiology..

[31] Ai T, Yang Z, Hou H, Zhan C, Chen C, Lv W (2020). Correlation of chest CT and RT-PCR testing in coronavirus disease 2019 (COVID-19) in China: a report of 1014 cases. Radiology..

[32] Rangaswami J, McCullough PA (2018). Heart failure in end-stage kidney disease: pathophysiology, diagnosis, and therapeutic strategies. Semin Nephrol.

